# Sphingosine-1-Phosphate Receptors Modulators Decrease Signs of Neuroinflammation and Prevent Parkinson’s Disease Symptoms in the 1-Methyl-4-Phenyl-1,2,3,6-Tetrahydropyridine Mouse Model

**DOI:** 10.3389/fphar.2020.00077

**Published:** 2020-02-21

**Authors:** Élise Pépin, Tim Jalinier, Guillaume L. Lemieux, Guy Massicotte, Michel Cyr

**Affiliations:** Groupe de recherche en signalisation cellulaire, Département de biologie médicale, Université du Québec à Trois-Rivières, Trois-Rivières, QC, Canada

**Keywords:** S1P receptors, FTY720, SEW2871, MPTP, neuroinflammation, neuroprotection

## Abstract

Sphingosine-1-phosphate (S1P) is a potent bioactive lipid mediator that acts as a natural ligand upon binding to five different receptors that are located in astrocytes, oligodendrocytes, microglial and neuronal cells. Recently, global activation of these receptors by FTY720 (fingolimod) has been suggested to provide neuroprotection in animal model of Parkinson’s disease (PD). Among S1P receptors, the subtype 1 (S1P1R) has been linked to features of neuroprotection and, using the selective agonist SEW2871, the present investigation assessed potential benefits (and mechanisms) of this receptor subtype in an established animal model of PD. We demonstrated that oral treatments with SEW2871 are able to provide protection to the same levels as FTY720 against loss of dopaminergic neurons and motor deficits in the 1-methyl-4-phenyl-1,2,3,6-tetrahydropyridine (MPTP) (30 mg/kg, i.p., 5 days) mouse model of PD. At the molecular level, we observed that the beneficial effects of both S1PR agonists were not associated with alterations in ERK and Akt levels, two markers of molecular adaptations in the striatum neurons. However, these compounds have the capacity to prevent signs of neuroinflammation such as the activation of astrocytes and glial cells, as well as MPTP-induced reduction of BDNF levels in key regions of the brain implicated in motor functions. These findings suggest that selective S1P1R modulation has the ability to provide neuroprotection in response to MPTP neurotoxicity. Targeting S1P1R in PD therapy may represent a prominent candidate for treatment of this neurodegenerative conditions.

## Introduction

Parkinson’s disease (PD) is a common age-related neurodegenerative condition, characterized by progressive loss of the nigrostriatal dopaminergic pathway and erosion of several neurological functions. Biochemical studies performed on *postmorterm* brains suggest that pathogenic factors most likely contributing to PD include a progressive neuroinflammatory reaction involving microglial activation and subsequent formation of pro-inflammatory cytokines such as the tumor necrosis factor (TNF-α) (Nagatsu and Sawada, 2007). Reduction in neurotrophins synthesis such as brain-derived nerve growth factor (BDNF) is another important feature associated with PD pathology (Shen et al., 2018). In support to this theory, several *in vitro* and *in vivo* studies propose that BDNF depletion occurring in various neuropathological conditions is mediated by the release of pro-inflammatory cytokines (Calabrese et al., 2014).

Although symptomatic improvement can be achieved by pharmacologically restoring dopaminergic transmission, the development of neuroprotective treatments that prevent or halt PD pathogenic processes is still awaiting. Emerging evidence have established that Fingolimod (FTY720), a non-selective sphingosine-1-phosphate receptors (S1PRs) modulator approved for the treatment of multiple sclerosis, can provide significant protection in mouse models of neurodegenerative conditions including two recent studies on PD (Aytan et al., 2016; Zhao et al., 2017). These later studies suggest that neuroprotective properties of FTY720 in a murine model of PD require direct effects of the drug on neuronal cells, which are presumably dependent on ERK activity. However, which specific S1PRs is responsible for these beneficial effects and whether non-neuronal mechanisms such as neuroinflammation are associated with brain damages is still unknown.

The present study will examine the efficacy of an oral treatment with the non-selective agonist FTY720 to subtype 1, 3, and 5 of S1PRs, or the selective agonist SEW2871 to subtype 1 of S1PRs (S1P1R), to prevent the 1-methyl-4-phenyl-1,2,3,6-tetrahydropyridine (MPTP) induced nigrostriatal loss and motor deficits in mice. In addition, potential mechanisms of action that include neuronal signaling, BDNF synthesis, and neuroinflammatory pathways will be investigated.

## Materials and Methods

### Animals

Twelve week-old male C57BL/6j mice (Charles River Laboratories, QC, CAN) were individually housed in a controlled room under a 14 h light/10 h dark cycle. Food and water were available *ad libitum*. All experiments were approved and carried out with the recommendations of the UQTR Institutional Animal Care and Use Committee (protocol #2016-MiC.24) in accordance with the Canadian Council on Animal Care.

### Pharmacological Treatments

**Figure 1:**
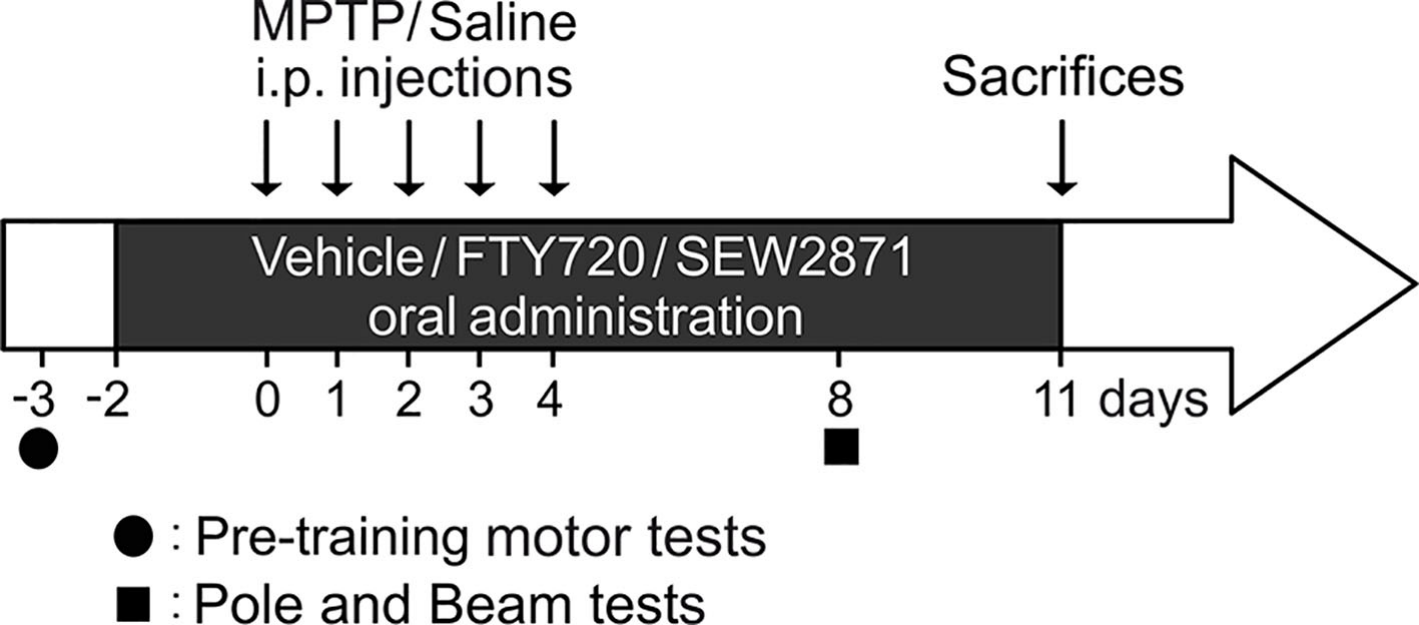
Experimental design. Three days before 1-methyl-4-phenyl-1,2,3,6-tetrahydropyridine (MPTP) injections, mice were pre-trained at the pole and beam tests by completing three trials within a day. Two days before MPTP injections, mice received an oral treatment with either vehicle [10% dimethyl sulfoxide (DMSO) and 25% Tween 20 v/v dissolved in saline 0.9% sodium chloride], FTY720 (1 mg/kg), or SEW2871 (20 mg/kg) once a day for a total of 14 days. At day 0, saline or MPTP (30 mg/kg/day) treatments were administered i.p. during 5 days. Motor abilities at the pole and beam tests were evaluated 8 days after the first MPTP injections. On day 11, all mice were sacrificed. The experimental setting involved six groups: 1) vehicle with saline treatments, *n* = 3; 2) FTY720 with saline treatments, *n* = 3; 3) SEW2871 with saline treatments, *n* = 3; 4) vehicle with MPTP treatments, *n* = 5; 5) FTY720 with MPTP treatments, *n* = 5; (6) SEW2871 with MPTP treatments, *n* = 5.

The experimental design is detailed in **Figure 1**. FTY720 and SEW2871 [5-(4-phenyl-5-trifluoromethylthiophen-2-yl)-3-(3-trifluoromethylphenyl)-1,2,4-oxadiazole] were purchased from Cayman Chemical (Ann Arbor, MI, USA) and MPTP hydrochloride from Toronto Research Chemicals (North York, ON, CAN). All mice (*n* = 24) were orally treated with either vehicle [10% dimethyl sulfoxide (DMSO) and 25% Tween 20 v/v dissolved in saline 0.9% sodium chloride], 1 mg/kg FTY720 (dissolved in vehicle) (Yazdi et al., 2015), or 20 mg/kg SEW2871 (dissolved in vehicle) (Dong et al., 2014) daily for 14 days. Mice were also intraperitoneally (i.p.) injected with saline or 30 mg/kg MPTP (dissolved in saline) (Xiao-Feng et al., 2016) once a day for five consecutive days. Our experimental groups were defined as follow: 1) vehicle + saline, *n* = 3 mice; 2) FTY720 + saline, *n* = 3; 3) SEW2871 + saline, *n* = 3; 4) Vehicle + MPTP, *n* = 5; 5) FTY720 + MPTP, *n* = 5; and 6) SEW2871 + MPTP, *n* = 5.

### Behavioral Assessments

Motor behavior was assessed using the pole and beam tests, as previously described (Chagniel et al., 2016; Bergeron et al., 2017). Briefly, at the pole test, mice were placed at the upper end of a vertical pole (diameter: 1.5 cm; length: 50 cm). The time required for each mouse to turn down and reach the base of the pole was recorded for three trials. The balance beam assessed two mouse abilities: the first one is the time taken to go across a narrow beam (width: 6 mm; length: 100 cm) to reach a dark goal box whereas the second one measured the stepping errors, i.e., foot-faults, occurring during the same trial. Mouse performances were recorded for three trials. A foot-fault was considered each time a paw fell under the beam midline. In all tests, mice were pre-trained three consecutive periods to remove the learning variables and a maximum time of 60 s was allowed to execute the tasks.

### Protein Levels Quantification

Mice were sacrificed by decapitation and brains were divided in half. The posterior part of the brain was immediately processed for immunofluorescence analyses. In the anterior part of the brain, we rapidly dissected out the striatum structure in both hemispheres and immediately freeze them on powdered dry ice. The striatum were pooled together and stored at −80°C until we performed protein extractions for Western blot analysis. Samples were homogenized in ice-cold radioimmunoprecipitation assay (RIPA) lysis buffer containing a cocktail of protease and phosphatase inhibitors (Roche, Indianapolis, IN, USA). Protein concentrations were quantified by Bradford assay (Bio-Rad, Hercules, CA, USA). Equal amounts of proteins (30–40 μg) were separated on sodium dodecyl sulfate/polyacrylamide gel electrophoresis (SDS/PAGE) gels and transferred onto nitrocellulose membranes. Immunoblots were performed overnight using the following primary antibodies: mouse monoclonal antibody against tyrosine hydroxylase (TH; 1:2,000; Millipore, Billerica, MA, USA), rabbit polyclonal antibody against dopamine transporter (DAT; 1:1,000; Millipore), rabbit polyclonal antibody against sphingosine 1-phosphate receptor 1 (EDG-1; 1:10,000; Abcam, Cambridge, MA, USA), rabbit polyclonal antibody against phospho-p44/42 MAPK (ERK1/2; Thr202/Tyr204; 1:1,000; Cell Signaling Technology, Whitby, ON, CAN), mouse monoclonal antibody against p44/42 MAPK (ERK1/2; 1:2,000; Cell Signaling Technology), rabbit monoclonal antibody against protein kinase B (p-Akt-Thr308; 1:1,000; Cell Signaling Technology), rabbit monoclonal antibody against total protein kinase B (Akt; 1:1,000; Cell Signaling Technology), rabbit polyclonal antibody against BDNF (1:400; Abcam), mouse monoclonal antibody against tumor necrosis factor alpha (TNF-α; 1:2,000; Abcam), mouse monoclonal antibody against glial fibrillary acidic protein (GFAP; 1:1,000; Cell Signaling Technology), and mouse monoclonal antibody against GAPDH (1:10,000; Abcam). Membranes were washed in tris-buffered saline (TBS)-Tween 0.1% and incubated with appropriate horseradish peroxidase-conjugated secondary antibody (1:5,000; Thermo Scientific, Ottawa, ON, CAN). To visualize protein bands, chemiluminescence reactions were performed (SuperSignal West Femto Chemiluminescence Kit, Pierce Chemical Co, IL, USA). Densitometry analysis were achieved using the VisionWorks LS software (UVP Bioimaging Upland, Upland, CA, USA) and expressed as relative optical density.

### Immunofluorescence Analysis

Posterior part of the brain was post-fixed overnight at 4°C in 4% paraformaldehyde in phosphate buffered saline (PBS), pH 7.5, and a few hours in 10% sucrose/4% paraformaldehyde (wt/vol). They were frozen in isopentane and stored at −80°C. Coronal brain sections (60 μm) containing the subtantia nigra pars compacta (SNc) and the ventral tegmental area (VTA) (−2.92 to −3.64 mm from the Bregma; Paxinos and Franklin, 2001) were sliced using a Leica CM3050S Cryostat (Leica, Richmond Hill, ON, Canada) and kept at 4°C in PBS. For free-floating immunofluorescence, sections were incubated in permeabilizing solution containing 1.2% Triton X-100 in PBS followed by blocking solution containing 10% normal goat serum in PBS to avoid non-specific binding. They were then incubated with primary antibodies: rabbit polyclonal antibody or mouse monoclonal antibody against TH (1:500; Millipore), mouse monoclonal antibody against neuronal nuclear antigen (NeuN; 1:200; Millipore), mouse monoclonal antibody against GFAP (1:300; Cell Signaling Technology), and rabbit monoclonal antibody against ionized calcium binding adaptor molecule 1 (Iba-1; 1:100; Abcam). The sections were rinsed in PBS and incubated in appropriate secondary antibody: goat anti-mouse conjugated with fluorescein isothiocyanate (FITC) or DyLight 594-conjugated goat anti-rabbit (1:500; Cell Signaling Technology), diluted in PBS containing 0.3% Triton X-100 and 2% next-generation sequencing (NGS) for 1 h at room temperature. After several washes in PBS, they were incubated with Hoescht 33342 (1:10,000/PBS, Invitrogen, Burlington, ON, CAN) for 15 min. Finally, the sections were rinsed several times in PBS and mounted in VECTASHIELD medium on Superfrost slides for visualization under a confocal spinning disk microscope (MBF Bioscience, Williston, VT, USA).

TH and NeuN positive cells were determined by unbiased stereological quantification using the optical fractionator of Stereo Investigator software (MBF Bioscience, Vermont, USA). Three coronal sections containing the SNc and VTA of both hemispheres were considered per animal: −3.16, −3.28, and −3.40 mm from the bregma. Borders of the SNc and VTA were defined using TH-immunostaining from a random starting point with 2X objective. Inside these borders, positive cells were counted with a 60X PlanApo oil-immersion objective and 1.4 numerical aperture attached to an Olympus BX51 microscope. A systematic sampling of the outlined area was made from a random starting point. Counts were recorded at predetermined intervals (x = 250, y = 150) and a counting frame (50x50 μm) was superimposed on the live image of each tissue section. Section thickness was measured by focusing on the top of the section, zeroing the z-axis, and focusing on bottom of the section (average section thickness was 60 μm with a range of 58.9–61.1 μm). The dissector height was set at 50 μm. Immunolabeled neurons were counted only if the first recognizable profile came into focus within the counting frame. This method certified a uniform, random, and systematic cell count. Focusing through the z-axis revealed that NeuN and TH antibodies penetrated the full depth of tissue sections. Positive cell counts were expressed as total number/mm^3^.

Semiquantitative optical densitometry measurements in the SNc and VTA structures were also conducted to evaluate Iba-1 and GFAP immunofluorescences. Three sections of both hemispheres were considered per animal: −3.16, −3.28, and −3.40 mm from Bregma. To delineate SNc and VTA, coronal midbrain sections labeled with Iba-1 or GFAP were co-immunostained with TH. Images of Iba-1 and GFAP immunolabeling were captured bilaterally with 2X objective using Olympus BX51 microscope and optical densitometry measurements were obtained using ImageJ software (NIH, USA).

### Data Analysis

The data were analyzed using the GraphPad Prism software (version 5.0, Graph Pad Software, San Diego, CA, USA) to perform one-way ANOVA followed by the Newman-Keuls *post hoc* tests. Data were reported as mean ± SEM. and statistical significance was set at *P* < 0.05.

## Results

### S1PRs Modulators Reduced MPTP-Induced Inflammation and BDNF Depletion Without Interfering With S1P1R in Striatum Homogenates

**Figure 2:**
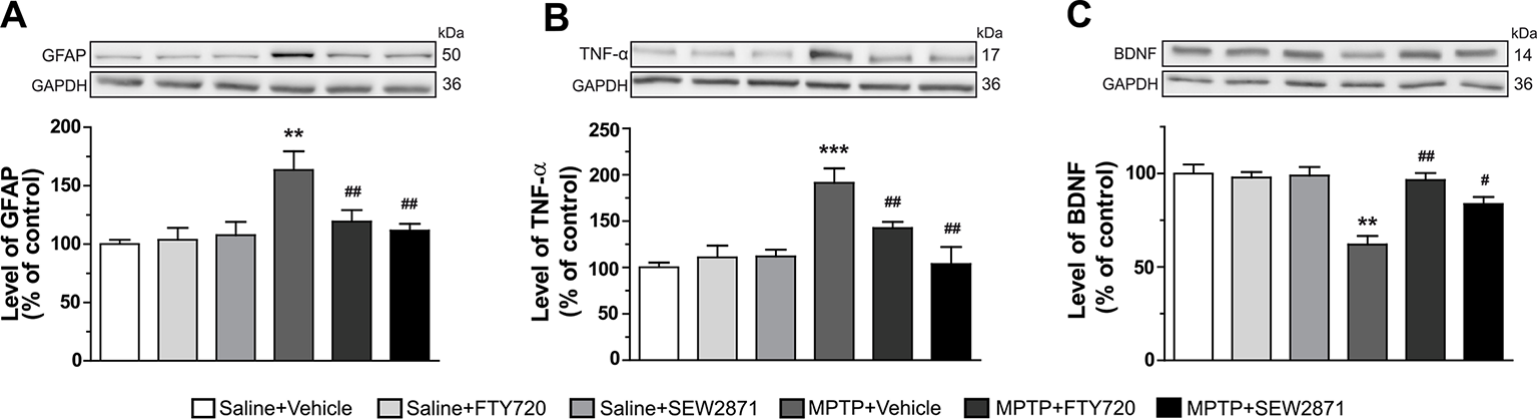
Levels of inflammatory markers are affected in the striatum. Levels of GFAP **(A)**, TNF-α **(B)**, and BDNF **(C)** were assessed by Western blot in the striatum of mice. The data, expressed relative to GAPDH, represent the mean of relative optical density in triplicate experiments of GFAP, TNF-α, and BDNF (expressed as a percentage of control values) ± S.E.M., *n* = 3–5 mice/group. **p < 0.01, ***p < 0.001 *vs*. vehicle + saline; #*p* < 0.05, ##*p* < 0.01 *vs*. vehicle + MPTP.

We first examined the manifestation of neuroinflammation in the striatum by the determination of GFAP and TNF-α levels using Western blot analyses. While the SEW2871 and FTY720 treatments alone have no effect, they were able to prevent the surge of striatal GFAP [**Figure 2A**; F(5,23) = 5.31, P < 0.01] and TNF-α [**Figure 2B**; F(5,23) = 8.20, P < 0.001] levels induced by MPTP. In parallel, expression levels of BDNF proteins have also been investigated and we observed a significant decrease of its protein levels after MPTP administrations, an effect that was totally prevented by SEW2871 and FTY720 treatments [**Figure 2C**; F(5,23) = 10.77, P < 0.01].

**Figure 3:**
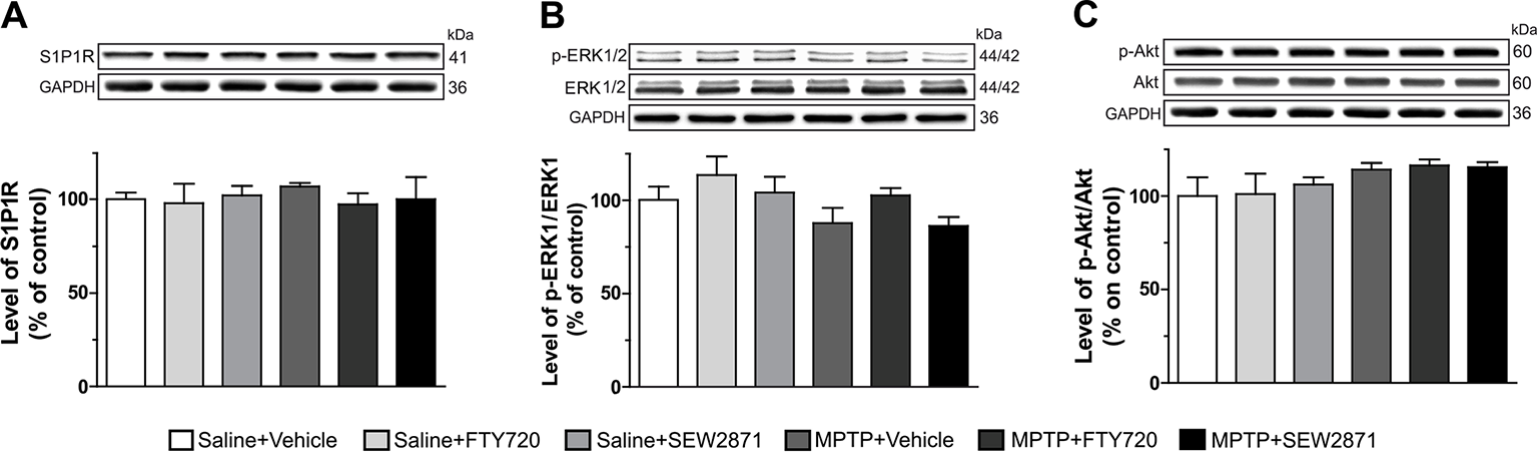
Levels of neuronal activity markers were unaltered in the striatum. Protein levels were evaluated by Western blotting with proteins extracted (30–40 μg of protein) from the mouse striatum. Levels of S1P1R **(A)**, phospho-Thr202-ERK1 **(B)**, and phospho-Thr308-Akt **(C)** were determined. GAPDH was used as loading control. Data represent the mean of relative optical density in triplicate experiments of S1P1R, phospho-Thr202-ERK1 and phospho-Thr308-Akt (expressed as a percentage of control values) ± S.E.M. Values are respectively expressed relative to GAPDH, total ERK, and total Akt, *n* = 3–5 mice/group.

The levels of S1P1R were estimated by Western blot analysis in the striatum of MPTP mice treated with the SEW2871 or FTY720. Statistical analyses did not revealed any difference between all groups [**Figure 3A**; F(5,23) = 0.20, P > 0.05]. Levels of phosphorylated ERK1/2 and Akt proteins were also investigated by Western blot in order to assess adaptive response to neuronal activation. We observed that in addition to total ERK1/2 and total Akt, levels of phospho-Thr202-ERK1 [**Figure 3B**; F(5,23) = 1.62, P > 0.05] and phospho-Thr308-Akt [**Figure 3C**; F(5,23) = 1.69, P > 0.05] were not altered by the different treatments.

### S1PRs Modulators Reduced MPTP-Induced Astrogliosis and Microgliosis in the SNc

**Figure 4:**
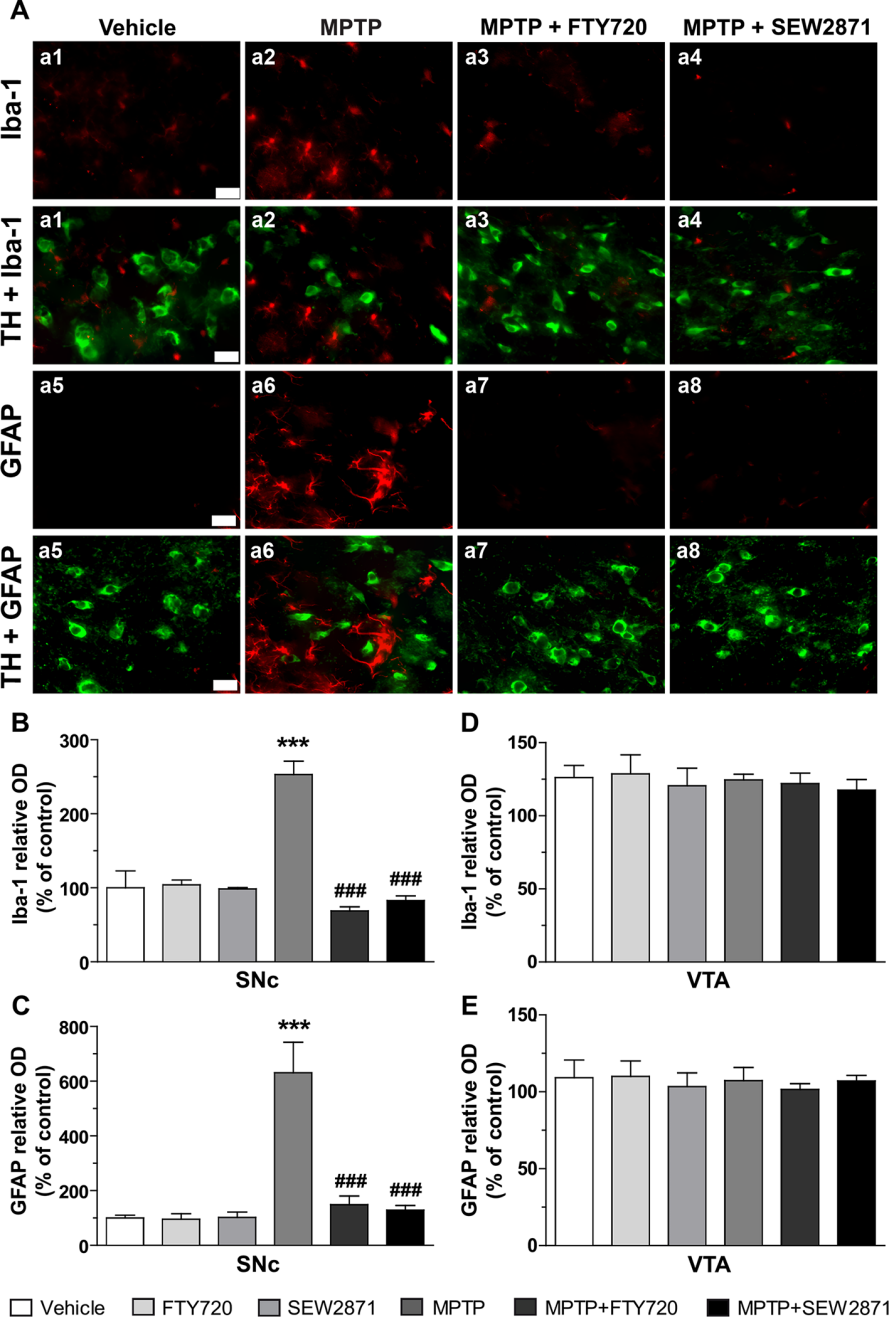
Signs of astrogliosis and microgliosis induced by 1-methyl-4-phenyl-1,2,3,6-tetrahydropyridine (MPTP) are prevented by S1P1R modulators. **(A)** Representative examples of Iba-1 immunoreactive microglia (panel a1 to a4; red color), GFAP-positive astrocytes (panel a5 to a8; red color) and TH-positive neurons (panel a1 to a8; green color) using epifluorescence microscope (40X objective) in the mouse subtantia nigra pars reticulate (SNr; −3.16 mm from the Bregma). Bar equals 50 µm. Fluorescence intensity of Iba-1 and GFAP were measured in the SNc **(B, D)** as well as in the ventral tegmental area (VTA) **(C, E)**. The data represent the mean of Iba-1 and GFAP relative optical density (expressed as a percentage of control values) ± S.E.M., *n* = 3–5 mice/group. ***p < 0.001 *vs*. vehicle + saline; ^###^ *p* < 0.001 *vs*. vehicle + MPTP.

Because MPTP injections were associated with signs of inflammation in the striatum, we next examined astrocytic and microglial activation in the two major midbrain DAergic centers, the SNc and VTA, using immunofluorescence technique. Astrocytic and microglial activation were revealed by GFAP and Iba-1 immunostaining, respectively. We observed the occurrence of faint immunostaining of Iba-1 positive microglia and GFAP positive astrocytes in the SNc of vehicle-treated mice (**Figure 4A**). However, in the SNc of MPTP-treated mice, an augmentation in the staining of Iba-1 as well as GFAP were observed, phenotypes known to be reminiscent of reactive microglia and astrocytes, respectively (**Figure 4A**). FTY720 and SEW2871 were able to prevent the occurrence of microgliosis and astrocytosis as the staining of Iba-1 and GFAP were similar to vehicle-treated mice (**Figure 4A**). In order to quantify these observations, we performed semi-quantitative optical densitometry measurements in the SNc and VTA of mice treated with MPTP and S1PR modulators. These analyses confirmed our qualitative observations and revealed that FTY720 and SEW2871 treatments prevented the robust increase of Iba-1 [**Figure 4B**; *F*_(5,23)_ = 48.66, *P* < 0.001] and GFAP [**Figure 4D**; *F*_(5,23)_ = 11.43, *P* < 0.001] staining in the SNc of mice after MPTP treatments. No effect of FTY720 and SEW2871 treatments alone were noticed in the SNc and no signs of reactive microglia [**Figure 4C**; *F*_(5,23)_ = 0.24, *P* > 0.05] or astrocytes [**Figure 4E**; *F*_(5,23)_ = 0.19, *P* > 0.05] were observed in the VTA following the different treatments.

### S1PRs Modulators Protects Against MPTP-Induced Nigrostriatal Cellular Loss

**Figure 5:**
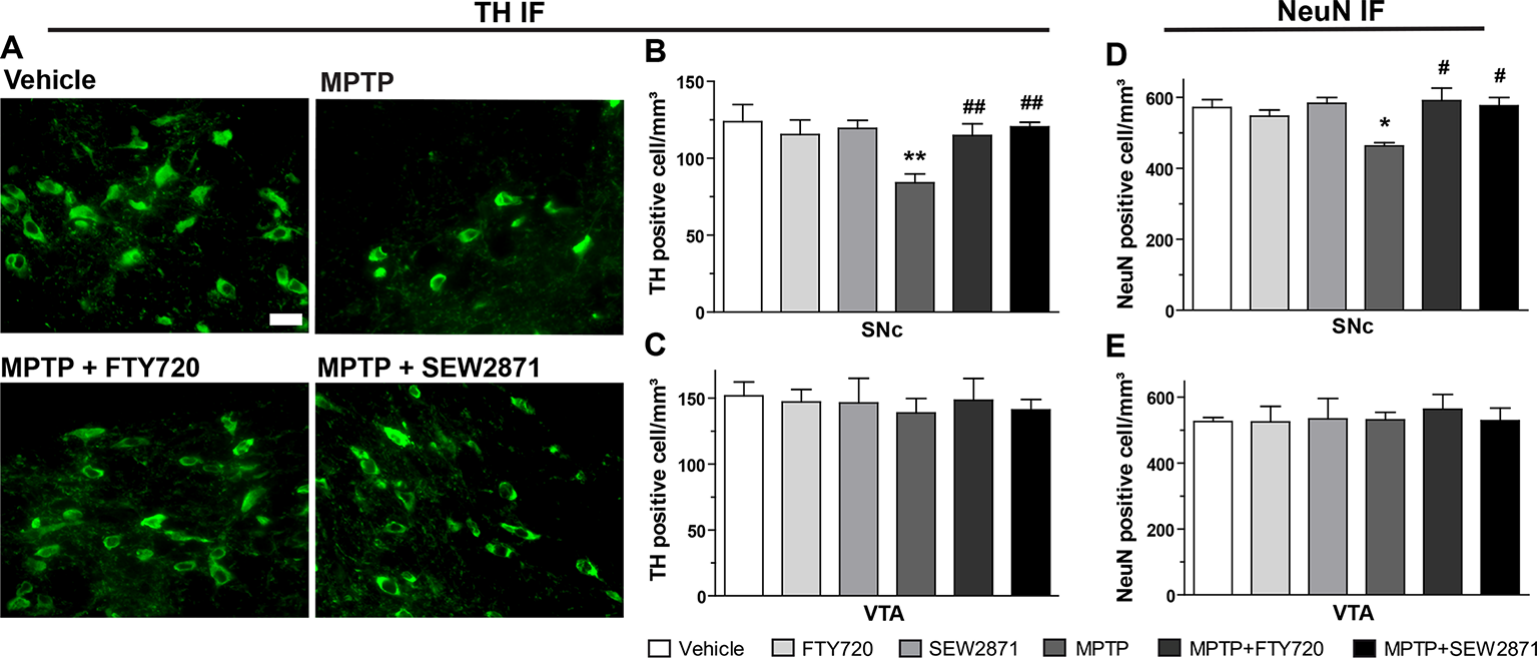
S1PR modulators protect against 1-methyl-4-phenyl-1,2,3,6-tetrahydropyridine (MPTP)-induced subtantia nigra pars compacta (SNc) cellular loss. Representative examples of tyrosine hydroxylase (TH)-immunostaining (green) using the 40X objective of the epifluorescence microscope in coronal sections (−3.16 mm from the Bregma) of mouse subtantia nigra pars reticulate (SNr). Bar equals 50 µm **(A)**. Stereological counts of TH- and neuronal nuclear antigen (NeuN)-positive neurons in the SNc **(B, D)** and ventral tegmental area (VTA) **(C, E)**. The data represent the mean of TH- and NeuN-positive cell numbers/mm^3^ (expressed as a percentage of control values) ± S.E.M., *n* = 3-5 mice/group. *p < 0.05, **p < 0.01 *vs*. vehicle + saline; ^#^ *p* < 0.05, ^##^ *p* < 0.01 *vs*. vehicle + MPTP.

The extent of MPTP-induced midbrain dopaminergic denervation was estimated by immunofluorescence techniques using two independent antibodies (**Figure 5A**), one raised against the neuronal marker NeuN and the second raised against TH. This rate-limiting enzyme for dopamine synthesis is widely used as a marker of dopaminergic depletion (Chagniel et al., 2012). In the SNc of MPTP-treated mice, our stereological method revealed a reduction in TH immunopositive cells that was totally prevented by the SEW2871 and FTY720 treatments [**Figure 5B**; *F*_(5,23)_ = 5.31, *P* < 0.01]. In contrast, no effect of either MPTP, SEW2871, or FTY720 administration was observed on TH immunopositive cells of the VTA [**Figure 5C**; *F*_(5,23)_ = 0.13, *P* > 0.05]. These data were replicated using the NeuN marker in the SNc [**Figure 5D**; *F*_(5,23)_ = 3.57, *P* < 0.05] and the VTA [**Figure 5E**; *F*_(5,23)_ = 0.15, *P* > 0.05].

**Figure 6:**
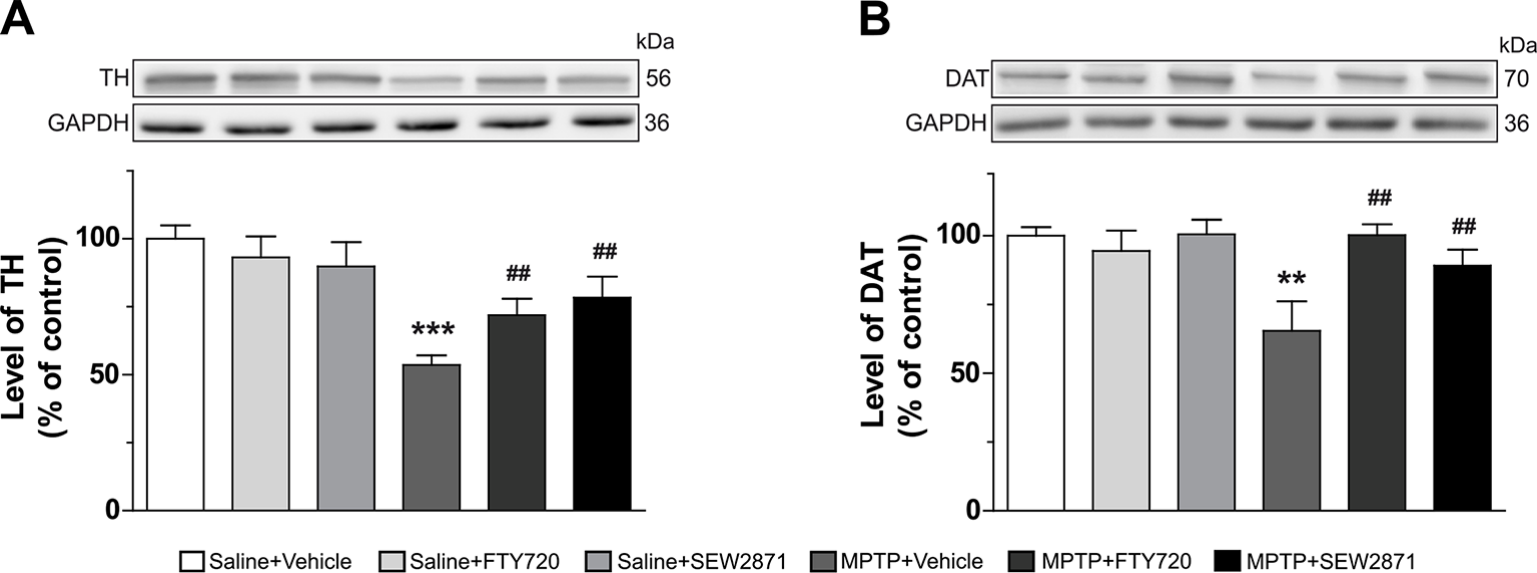
Dopamine terminals were spared in the striatum following S1P1Rs modulators treatments. Levels of tyrosine hydroxylase (TH) **(A)** and dopamine transporter (DAT) **(B)** were determined in the mouse striatum by Western blot experiments. The data, expressed relative to GAPDH, represent the mean of relative optical density in triplicate experiments of TH and DAT (expressed as a percentage of control values) ± S.E.M., *n* = 3–5 mice/group. **p < 0.01, ***p < 0.001 *vs*. vehicle + saline; ^##^ *p* < 0.01 *vs*. vehicle + MPTP.

In addition to the VTA and SNc, we also investigated the detrimental effects of MPTP on striatal dopamine terminals by using Western blot analysis. Two well-known markers of dopaminergic neuron terminals were employed and robust decreases in TH [**Figure 6A**; *F*_(5,23)_ = 5.92, *P* < 0.001] and DAT [**Figure 6B**; *F*_(5,23)_ = 3.98, *P* < 0.01] levels were observed after MPTP injections. SEW2871 and FTY720 administrations were both able to prevent this effect of MPTP (**Figures 6A, B**).

### Motor Abilities Were Preserved With FTY720 and SEW2871 Administrations

**Figure 7:**
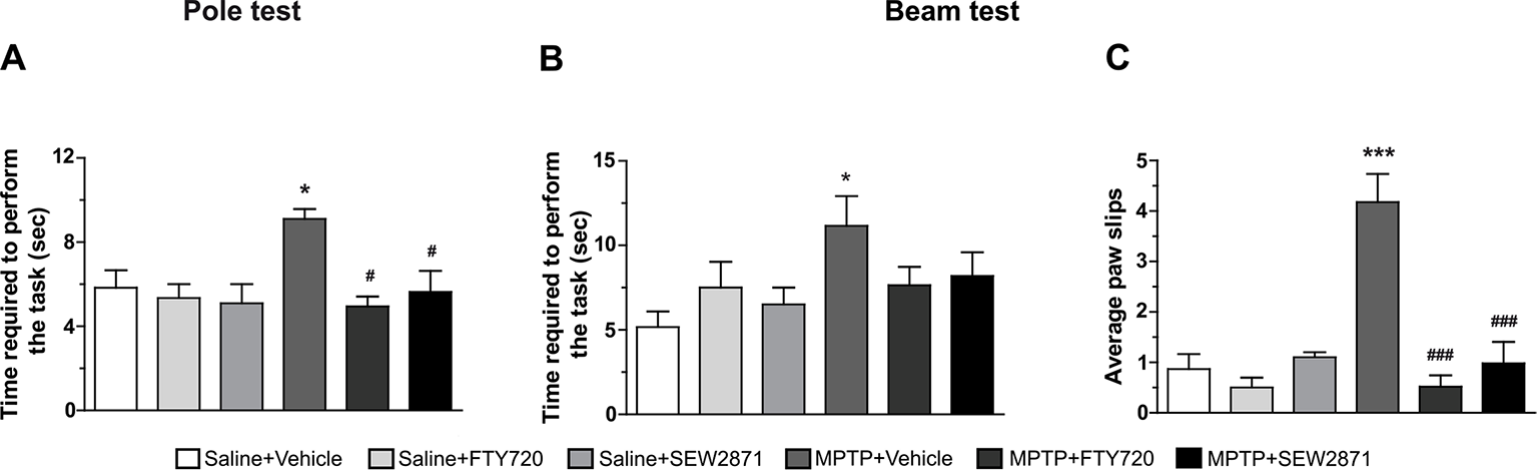
Motor behavioral outcomes. Motor abilities on the pole and beam tests were evaluated. Data represent the mean time require to perform the pole **(A)** and beam **(B)** tests and the mean number of foot-faults occurring during the beam test **(C)** ± S.E.M., *n* = 3–5 mice/group. *p < 0.05, ***p < 0.001 *vs*. vehicle + saline; ^#^ *p* < 0.05, ^###^ *p* < 0.001 *vs*. vehicle + MPTP.

To assess the effect of treatments on motor abilities, we performed two behavioral tests, namely the pole and beam tests. In particular, the pole test estimated bradykinesia and motor coordination whereas the beam test analyzed skilled walking and overall coordination. It is noteworthy that mice were pre-trained in order to remove the learning variables associated to these tests. We observed that while SEW2871 or FTY720 treatments alone were without effects on motor behaviors, they prevented the MPTP-induced motor deficits in all tasks. We observed impaired performances of MPTP treated mice at the pole test [**Figure 7A**; *F*_(5,23)_ = 3,47, *P* < 0.05] as well as an increase in the time taken to go across the beam [**Figure 7B**; *F*_(5,23)_ = 2,81, *P* < 0.05] and in the number of stepping errors at the beam test [**Figure 7C**; *F*_(5,23)_ = 13,48, *P* < 0.001]. The time required to perform the pole test and the beam test, as well as the average foot-faults were returning to control values when MPTP mice received either SEW2871 or FTY720 administrations. Note that statistical significance was not reached for the time required to perform the beam test in both groups.

## Discussion

The present study demonstrates that oral treatments with FTY720 and SEW2871 provide protection against loss of dopaminergic neurons and motor deficits in a mouse model of PD. In addition to PD, FTY720 was known to exert several beneficial pharmacological effects on central nervous system (CNS) cells contributing to the treatment of various neuropathological conditions, such as stroke (Brunkhorst et al., 2014), Huntington’s (Di Pardo et al., 2014), and Alzheimer’s diseases (Martin and Sospedra, 2014). However, this study is the first to demonstrate such CNS positive outcomes for the S1P1R-selective agonist SEW2871. In addition, we provide evidence that FTY720 and SEW2871 treatments have the capacity to prevent activation of astrocytes and glial cells, as well as prevent the decrease of BDNF levels, in regions of the brain involved in the control of motor functions. Our finding propose that drugs acting *via* the S1P1R have the capacity to provide neuroprotection to the detrimental effects of MPTP treatments.

At this point it is important to emphasize that the MPTP treatment (5 days, 30 mg/kg, once daily) used in this mouse study is a well validated model and that the results obtained in our experiments are in line with previous observations (Jackson-Lewis et al., 2012; Wang et al., 2015). The loss of nigrostriatal dopaminergic neurons is parallel by a robust increase of glial and astrocyte reactivity in the SNc and striatum, in addition to impaired motor behavior. It is interesting that no alteration at the striatal levels of phosphorylated ERK and Akt, two markers of molecular adaptations of striatal spiny projection neurons, are observed after MPTP treatments. This finding was somewhat expected as variation in the activity of these striatal kinases is associated with high dopaminergic depletion; observed for instance in unilateral 6-hydroxydopamine (OHDA) treated mice (Zhao et al., 2017) or more severe MPTP mouse model (Motyl et al., 2018). In our study, the degree of dopaminergic depletion is of 50%, as evaluated by TH levels in the striatum of MPTP mice, which is reminiscent of a mild dopaminergic-depletion. Limitations of MPTP administrations as a model of PD has been well documented previously (Bezard et al., 2013). Part of the problem with the MPTP toxin models is their acute nature, which is completely different from the insidious progression of PD observed in patients. Compensatory changes may arise in patients over the course of the disease that would not have an opportunity to occur in the acute animal models. In addition, PD occurs most frequently in elderly patients, usually around the age of 60 or older. Unfortunately, because of the inconvenience and cost of housing the animals for an extended period our study did not use older animals. In addition, a closer look at the differences in behavior, physiology, and gene expression between rodents and humans partially reveals why the animal studies do not translate well to clinical studies. With that said, while it may not be realistic to obtain a single animal model that completely reproduces every feature of a human disease as complex as PD, it would still be interesting to verify whether FTY720 and SEW2871 provide also protection during the formation of α-synuclein aggregates that resemble Lewy bodies or Lewy neurites. For instance, chronic administration of neurotoxins induces progressive PD rodent models that include α-synuclein aggregates in addition to motor deficits and rapid nigrostriatal dopaminergic cell death (Creed and Golberg, 2018). Furthermore, genetic-based approaches including transgenic models, viral vector-mediated models based on genes linked to monogenic PD as well as introduction of α-synuclein preformed fibrils all induce toxic protein aggregates in rodents. It would have been interesting to investigate the effects of FTY720 and SEW2871 in these particular models or in a combination of models to study the interplay between genetics and environment in an attempt to untangle the heterogeneity and mechanisms underlying PD.

One striking original finding in our study is the demonstration that SEW2871 and FTY720 prevent the occurrence of neuroinflammatory signs associated with MPTP treatments in mice. In particular, we establish that the SEW2871 and FTY720 treatments have the capacity to prevent the robust increase in GFAP and TNF-α expression levels in the striatum as well as the increased expression of GFAP and Iba-1 of the SNc observed in the MPTP treated mice. While the mechanism of action is still unclear, some multiple sclerosis studies performed *in vitro* and in animal models have demonstrated that FTY720 reduced microglia mediated inflammation and also diminished astrocyte activation in association to the neuronal protection (Choi et al., 2011; Miguez et al., 2015; Rothhammer et al., 2017; O'Sullivan et al., 2018). Whether the ongoing astrocytosis and microgliosis in the brain of our MPTP treated mice is responsible for the dopamine neuronal loss is unknown, this question is still under extensive debate in PD field (Kaur et al., 2017). However, in the VTA, a region where no effect of the MPTP treatment on dopaminergic neurons is noticed, no signs of astrocytosis or microgliosis are observed. This is at least one evidence showing that neuroinflammatory signs are selectively detected in brain regions where neuronal death is observed (Mosharov et al., 2009). Our data support a new role for S1P1R agonists in mediating anti-inflammatory effects in the MPTP mouse model of PD.

Treatments with FTY720 and SEW2871 did not alter the levels of phosphorylated ERK or Akt in the striatum of MPTP treated mice. In model cell lines transfected with the S1P1R subtype, the natural ligand S1P, FTY720, and SEW2871 appear to activate ERK and Akt pathways (Jo et al., 2005). In mouse models of PD, two recent studies have respectively reported that FTY720 increases levels of p-ERK or p-Akt in the striatum of 6-OHDA or MPTP treated mice (Zhao et al., 2017; Motyl et al., 2018). Again, the mild dopaminergic depletion in our MPTP mice may be responsible for the divergence with previous findings. Another interesting possibility is the fact that FTY720 and SEW2871 may have different effects on S1P1 expression on neuronal *versus* glial cells. However, we believe that this particular question would necessitate further research as only few studies have addressed this topic and the effect of SEW2871 is not assessed *in vivo* or in the context of PD. It is however clear from our data that activation of ERK and Akt pathways would play a minor role in the beneficial effects of FTY720 and SEW2871 on dopamine neuronal survival and neuroinflammation. On the other hand, both treatments have the capacity to prevent the reduction of BDNF levels observed in the striatum of MPTP mice. The link between FTY720, increased BDNF levels and neuroprotection has been well described in cell cultured and animal models of Rett syndrome (Deogracias et al., 2012), HD (Miguez et al., 2015), AD (Doi et al., 2013; Fukumoto et al., 2014) and PD (Giasson et al., 2002). For instance, in cultured neurons, FTY720 increases BDNF levels and counteracts N-methyl-d-aspartate (NMDA)-induced neuronal death in a BDNF-dependent manner (Di Menna et al., 2013; Cipriani et al., 2015). In PD animal models, BDNF treatment has been shown to reduce the loss of dopaminergic neurons (Tsukahara et al., 1995; Levivier et al., 1995). Our findings are suggesting that the decrease of BDNF levels may be one contributing factor responsible for the observed dopaminergic neuronal loss, and that rescuing this deficit may underlies the protective effect of FTY720 and SEW2871 treatments.

Despite that our study raised the interesting possibility that prevention of neuroinflammation and striatal BDNF levels recovery, rather than striatal Akt and ERK signaling modulation, are responsible for the neuroprotective effects of FTY720 and SEW2871 in the MPTP mouse model, the exact target mechanisms remain to be determined. For instance, we cannot exclude that brain inflammation could rely on peripheral mechanisms. Yang et al. (2018) demonstrate that lymphocyte infiltration contributes to brain neurodegeneration in the MPTP mouse model. On that line and, given the ability of FTY720 and SEW2871 to produce lymphopenia (Sanna et al., 2004; Morris et al., 2005), we cannot exclude that the neuroprotection we observed from these drugs in the MPTP-treated mice is dependent on reduced neuroinflammatory processes subsequent to peripheral mechanisms. Administration of S1P modulators after MPTP exposure would have help separating out an effect of drugs on peripheral immune cell recruitment and direct glial modulation. Of course, the precise contributions of central and peripheral mechanisms to the benefit of FTY720 and SEW2871 in PD pathology warrants further investigations. On the other hand, our data suggest that the anti-inflammatory effects of FTY720 and SEW2871 may be responsible for rescuing BDNF levels in the striatum of MPTP-treated animals. This scenario is consistent with the recent literature indicating that production of TNFα and subsequent activation of TNFα receptor 1 can be responsible for down-regulating BDNF expression in the hippocampus. Precisely, Liu et al. (2017) demonstrated that hippocampal BDNF expression is markedly reduced in mice after peripheral nerve injury, an effect that was totally abolished following genetic deletion of TNFα receptor 1. In addition, it has been documented that BDNF is synthesized not only in neurons, but also in astrocytes (Saha et al., 2006). It is therefore tempting to propose the interesting possibility that astrocytes are also mediating these effects given that S1PR expression are enriched in these cells in addition to their capacity to produce BDNF. It would be of interest to find out to what extent the beneficial effects on BDNF expression exerted by FTY720 and SEW2871 are dependent on S1PR and TNFα receptor activation in the striatum of MPTP-treated mice.

Our results show that similar beneficial effects observed with either SEW2871 or FTY720 treatments in the MPTP mouse model of PD suggest a crucial role for the subtype 1 of S1PRs. The specificity of SEW2871 to bind to S1P1R is undeniably well recognized (Bolick et al., 2005; Blaho and Hla, 2014). On the other hand, FTY720 is less selective to that particular S1PR. FTY720 need to be phosphorylated *in vivo* by sphingosine kinase-2 to form the active moiety FTY720-phosphate known to bind subtypes −1, −3, −4, and −5 (Brinkmann et al., 2002; Soliven et al., 2011). At the exception of S1PR4, all S1PRs subtypes are expressed in the central nervous system and S1P1R and three levels are substantially high in the brain (MacLennan et al., 1994; Zhang et al., 1999). Interestingly, S1P1R is located on astrocytes, oligodendrocytes, microglial and neuronal cells throughout the brain, including region of the brain associated with the control of motor function (Chae et al., 2004; Aktas et al., 2010). Notably, S1P1R responses in astrocytes has been well studied in human and rodent studies (Healy et al., 2013; Wu et al., 2013). For instance, our finding confirmed S1P1R protein expression in the striatum structure. Whether S1P1R is exclusively responsible for the neuroprotection we report using SEW2871 or FTY720 treatments is undetermined, but some evidence are supporting this contention in the literature. For instance, the role of S1P1R in the beneficial action of FTY720 is reported in animal models of stroke and PD (Dev et al., 2008; Hasegawa et al., 2010; Imeri et al., 2014; Brunkhorst et al., 2014; Sun et al., 2016; Zhao et al., 2017; Motyl et al., 2018). These studies all demonstrate that the effect of FTY720 is largely mediated by the S1P1R.

Consistent with other investigations, we observed that FTY720 could exert beneficial effects in PD mouse model (Zhao et al., 2017; Motyl et al., 2018). This drug has been shown to exert several pharmacological effects on CNS cells which may contribute to the treatment of various neuropathological conditions, such as stroke (Brunkhorst et al., 2014) Huntington’s (Di Pardo et al., 2014) and AD (Martin and Sospedra, 2014). In that line, it was recently found to reduce the density of pathological plaques and decreased the number of pro-inflammatory cells in animal models of AD (Fukumoto et al., 2014; Aytan et al., 2016). Unfortunately, both *in vitro* and *in vivo* experiments have demonstrated that FTY720, accumulating above a certain threshold in the brain, becomes less effective (Aytan et al., 2016) and even neurotoxic (van Echten-Deckert et al., 2014). In fact, studies have documented the possibility that FTY720 can alter normal brain physiology *via* a mechanism involving hyperphosphorylation of Tau proteins (Attiori Essis et al., 2015).

In conclusion, we observed that pharmacological targeting of S1P1R with the specific agonist SEW2871 conferred strong resistance to MPTP-induced dopaminergic depletion, inflammation processes and motor dysfunctions. From a clinical perspective, it seems thus plausible that selective activation of S1P1R by SEW2871 (or other chemical analogs) might be more effective and probably safer, knowing that this drug is not prone to induce Tau hyperphosphorylation (St-Cyr Giguere et al., 2017). In addition, many adverse events, including hypertension, macular edema, pulmonary toxicity, and hepatotoxicity, have been associated with FTY720, because of its off-target interactions with other S1PRs subtypes, particularly with S1PR3. The activation of S1PR3 by FTY720 was at least partially responsible for its side effects on the cardiovascular system and organ fibrosis, which may cause prominent safety issues (Cohen and Chun, 2011). Several drugs acting more selectively on S1PRs subtypes have been developed in recent years (Guerrero et al., 2016) and future experimentation are required to test for there efficiencies and safeties in the context of PD.

## Data Availability Statement

All datasets generated for this study are included in the article.

## Ethics Statement

This study was carried out in accordance with the recommendations of the UQTR Institutional Animal Care and Use Committee and performed in accordance with the Canadian Council on Animal Care.

## Author Contributions

ÉP and MC designed the study. ÉP conducted this research. TJ and GL contributed for the behavioral experiments. ÉP, MC, and GM wrote the manuscript. MC and GM contributed to the conceptual frame of the study and edited the manuscript.

## Funding

This work was supported by the Natural Sciences and Engineering Research Council of Canada (Grant #2017-06411). EP is the holder of a Fonds de recherche du Québec–Santé (FRQS) studentship.

## Conflict of Interest

The authors declare that the research was conducted in the absence of any commercial or financial relationships that could be construed as a potential conflict of interest.
